# Exploring HIV risks, testing and prevention among sub-Saharan African community members in Australia

**DOI:** 10.1186/s12939-018-0772-6

**Published:** 2018-05-21

**Authors:** Amy B. Mullens, Jennifer Kelly, Joseph Debattista, Tania M. Phillips, Zhihong Gu, Fungisai Siggins

**Affiliations:** 1 School of Psychology and Counselling, Institute for Resilient Regions, University of Southern Queensland, Ipswich Campus, 11 Salisbury Road, Ipswich, Qld 4305 Australia; 2 School of Health and Wellbeing, University of Southern Queensland, Ipswich Campus, 11 Salisbury Road, Ipswich, Qld 4305 Australia; 3 Queensland Health, Metro North Public Health Unit, Bryden Street, Windsor, Qld 4030 Australia; 4 Ethnic Communities Council of Queensland, PO Box 5916, West End, Qld 4101 Australia; 5 Kalpa purru Wirranjarlki, Anyinginyi Health Aboriginal Corporation, 1 Irvine Street, PO Box 40, Tennant Creek, NT 0861 Australia

**Keywords:** CALD, PREP, HIV screening, African community members, HIV risks

## Abstract

**Background:**

Significant health disparities persist regarding new and late Human Immunodeficiency Virus (HIV) diagnoses among sub-Saharan African (SSA) communities in Australia. Personal/cultural beliefs and practices influence HIV (risk, prevention, testing) within Australia and during visits to home countries.

**Method:**

A community forum was conducted involving 23 male and female adult African community workers, members and leaders, and health workers; facilitated by cultural workers and an experienced clinician/researcher. The forum comprised small/large group discussions regarding HIV risk/prevention (responses transcribed verbatim; utilising thematic analysis).

**Results:**

Stigma, denial, social norms, tradition and culture permeated perceptions/beliefs regarding HIV testing, prevention and transmission among African Australians, particularly regarding return travel to home countries.

**Conclusions:**

International travel as a risk factor for HIV acquisition requires further examination, as does the role of the doctor in HIV testing and Pre-exposure Prophylaxis (PrEP). Further assessment of PrEP as an appropriate/feasible intervention is needed, with careful attention regarding negative community perceptions and potential impacts.

## Background

Rates of new Human Immunodeficiency Virus (HIV) notifications and late HIV diagnoses in Australia are significantly higher among members of specific culturally and linguistically diverse (CALD) communities, in particular among sub-Saharan Africans (SSA) [1]. In 2016, SSA HIV notification rates (predominantly heterosexual), were 10.9 per 100,000 compared to 3.4 per 100,000 in Australian born populations [1]. Further, the proportion of late HIV diagnoses (defined as a CD4+ cell count below 350 cells/ul) was high among people born in SSA (43%), suggesting significant delays between infection and access to appropriate testing/treatment services [1]. Of note, approximately 63% of those from SSA arrived in Australia within the previous 5 years, suggesting HIV acquisition prior to arriving in Australia [1]. Pre-migration HIV acquisition and HIV transmission during return visits to home countries (post-migration) may be a significant issue in terms of being underestimated [2, 3], and may be associated with low HIV health literacy, uncertainty and fears over testing and limited awareness of novel prevention strategies [3]. However, this may also be associated with delayed testing and risks for transmission post-settlement [4, 5].

Sub-Saharan African migrants have been identified as a priority group for targeted HIV prevention/treatment in Australia [6]. Over time, migrants who have established themselves in Australia may choose to return to their country of origin for short periods due to business, family or personal reasons. The frequency of travel to high prevalence regions has been recognised as an associated risk factor for HIV transmission [7, 8]. As such, travellers have been referred to as a high risk ‘bridge’ population [3, 7], exposed not only to a range of HIV risks within a high prevalence setting, but with the additional risks that may accompany those whom travel recreationally or for business [8–10].

Studies suggest that personal and cultural beliefs, and cultural practices and traditions, can strongly influence health behaviours including sexual relationships, sexual health and associated risk reduction strategies such as condom use [7, 11–16]. A range of such beliefs and practices, overlayed by social and economic pressures finds expression in sexual behaviours, such as multiple and concurrent partnerships, and may occur more frequently among some SSA [17, 18]. Mah and Halperin [17] postulate multiple and concurrent sexual partners are a “deeply rooted social and cultural phenomenon” (pp. 15) in SSA people. Further, the cultural practice of polygamy allows a man to have multiple wives [19]. Such social and cultural practices are increasingly associated with increased risks for HIV transmission [14, 17–21], however, Sawers [22] suggests biomedical determinants such as sexually transmitted coinfections and inflammatory or ulcerating pathology are more significant factors for increased HIV transmission in SSA.

The nature of HIV risk exposure among SSA migrants living in Australia and when travelling to home countries is not well understood. The literature suggests a number of cultural practices (sexual and non-sexual) that may increase risk of HIV acquisition and transmission, such as ‘widow cleansing’ and inheritance after the death of a husband [7, 15, 23–25], and initiation rites of young girls at first menstruation [26]. These sexual cultural practices are reported to occur in Botswana [7], Kenya [23, 24], Malawi [15], Mozambique [11], Tanzania [11, 23], Uganda [25], and Zambia [7, 26]. There is a belief that sexual rituals are sacred and protected, consequently individuals may perceive they are not vulnerable to HIV [11, 23] when they may be at increased risk.

Non-sexual HIV risks may occur through the use of shared unsterilized knives, razor blades and other cutting instruments, utilised for male and female circumcision, tribal markings [7], and personal grooming [15, 23]. Other non-sexual HIV exposure may occur through the re-use of unsterilized needles for injections and blood transfusions [8, 23]. Therefore, migrants returning to their home country may become exposed to HIV by having their hair cut, shaving, or if they become ill and require a blood transfusion or injection [27, 28].

Negative attitudes towards condom use has also been reported within SSA [7], including the misconception that HIV can pass through condoms or that condom use can infect a person with HIV [15, 29, 30]. Furthermore, SSA female migrants in Australia have reported unfavourable attitudes towards condom use, for example condoms are unnatural or ‘spoil’ sex, and perceived their partners do not like using them [30]. Uwah and Ebewo [7] suggest condoms are seldom used by African community members who follow more traditional practices, where the importance of having children may outweigh risks regarding HIV.

Pre-exposure Prophylaxis (PrEP) has been identified as a potentially valuable prevention strategy among higher risk populations [31–35] particularly as an intervention for vulnerable people who may experience barriers to negotiating condom use with sexual partners [13]. However, despite a number of ongoing PrEP pilot studies across several states focussing on men who have sex with men, and increasing community use of PrEP among this group [36], little is known about the acceptability, feasibility and cultural appropriateness of PrEP among CALD populations, generally and in particular SSA migrants [6].

To identify potential risks for HIV infection amongst Queensland SSA community members in Australia a community forum of key stakeholders was facilitated. The objective of the forum was to provide an opportunity for community leaders and workers to discuss concerns about the nature and level of HIV risk exposure and behaviour within Queensland SSA communities. Participants were also invited to describe the nature and level of risk for HIV infection among members travelling between Australia and Africa and the potential for PrEP to be utilised as an acceptable prevention strategy.

## Methods

This project was developed primarily as a forum for community workers, stakeholder and leaders to receive and share information and experiences regarding HIV (level and nature of risks within Australia and during return visits to Africa).Additionally, this project sought to gather information about the acceptability, and accessibility of HIV testing and PrEP for members of the Queensland SSA community within Australia and during return visits to home SSA countries. As an opportunity for community based research, the project employed a Qualitative Descriptive Design [37] to explore risks associated with the transmission of HIV. Qualitative descriptive research is used frequently for health related studies where the everyday events of individuals or groups is being described in terms of the participant’s views or intended meaning. A qualitative descriptive approach is ideal when a meaningful description of a phenomenon is preferred and needed [38]. Descriptive studies gather data in order to describe events [39] therefore, given the dual nature of this forum as a community based provision and collection of information, qualitative descriptive research was considered most suited to this project which sought to describe what is known about HIV prevention and transmission among SSA community members.

### Procedures

A community forum was conducted to explore perceptions regarding HIV prevention, transmission, and other contemporary HIV issues such as, PrEP, Post-exposure Prophylaxis (PEP), Treatment as Prevention (TasP) and testing among SSA community leaders and key stakeholders. The forum employed focus groups to gather data that could lead to an increased understanding regarding HIV infection and HIV risks, including protective strategies within Australia and during return visits to SSA.

Purposive sampling was employed to recruit participants, as this method of recruitment enables the deliberate selection of specific individuals who can supply critical and relevant information [40]. Additionally, purposive sampling enables in-depth information gathering from smaller, targeted groups [41]. Therefore, those involved in this study needed to be familiar with African cultural practices and health issues, possess English fluency and be willing to attend a small group forum. Staff from a non-government organisation (NGO) with expertise in sexual health service delivery invited SSA community leaders and members, and community and health workers (whom have familiarity of working with African community members) to discuss views held by SSA community members. Participants were selected by the NGO to participate in the workshop for their community knowledge, networking and expertise through targeted invitations. Recruitment involved NGO staff members sending invitees a flyer asking them to attend a special forum to discuss new ideas and strategies for HIV prevention in their communities. A Saturday morning was chosen to ensure attendance was not impeded due to work commitments and the forum was held at a community venue known to attendees.

Verbal consent was considered culturally appropriate due to sensitivities associated with the topic areas and consistent with other community-based sexual health research [42] and participants agreed to participate in the project and subsequent evaluation if they attended the forum. Participants completed a registration form (including their name and contact details if they wished to be contacted regarding future similar community forums). At the commencement of the forum, participants were welcomed and provided with a further verbal explanation about the community forum, stressing that participation was voluntary and that responses would be non-identified and aggregated into a group summary for the purposes of future health planning and evaluation. The Sexual Health Manager of the NGO, an experienced Public Health coordinator from a major metropolitan health district, and a researcher from a university (a registered clinical/health psychologist, with a background in sexual health), provided brief and culturally-specific and relevant information about the forum. Participants were requested to remain respectful of other participants’ views (and to maintain confidentiality regarding contributions) prior to focussed discussions and data collection. Ethics approval was granted by the University of Southern Queensland Human Research Ethics Committee (H16REA232) to allow for the formal analysis and dissemination of information collected during the community forum.

### Data collection

The forum consisted of a combination of short presentations (10–20 min) on four topics, by members of the project team whom have topic expertise. The forum topics included: (a) HIV transmission and risks, (b) epidemiology, (c) prevention approaches, and (d) PrEP. Small group discussions were initially held at roundtables following each presentation and later, large focussed group discussions were conducted using pre-established trigger questions. Discussion topics focused on: [1] general risks, [2] risks associated with travelling back home to Africa for visits, [3] prevention strategies, and [4] perceived effects of an intervention strategy (PrEP). Each group discussion included a facilitator (a member of the research team) and another research team member who served as a scribe. Audio recording was not undertaken due to anonymity and confidentiality concerns, cultural appropriateness and audibility concerns. Semi-structured questions included in the forum discussion are outlined in Table 1, consistent with the project aims.

**Table 1:** Topics and semi-structured questions

Discussion topics	Semi-structured discussion questions
Risks in Australia	Who is most at risk in the SS African communities? What behaviours put them at higher risk?
Risks travelling to home countries	Who is most at risk, when travelling back home? Why are they at higher risk?
Prevention approaches	What is working to prevent HIV, Here? In home countries? Past? Current? How has HIV prevention been promoted?
PrEP^a^ acceptability and feasibility	Do you think PrEP would be acceptable in your community? What are the barriers for using PrEP?

^a^PrEP = Pre-exposure prophylaxis

### Data analysis

Transcripts were prepared detailing the responses from participants to key discussion questions raised at the forum. The content of each transcript was analysed to explore forum participants’ perceptions of HIV risks and approaches to prevention in Australia and when returning on visits to home countries. Thematic analysis was employed to analyse the data as this approach provided a means of interpreting the data by identifying and understanding common patterns that emerged from participants or focus groups [39]. Thematic analysis necessitated becoming immersed in the data through reading and rereading each transcript multiple times in order to identify common aspects that emerged from the data [39]. A matrix chart was used to illustrate common aspects such as repeated concepts and patterns in the transcripts. Employing this manual coding technique enabled major themes and sub-themes to be identified. Further, two researchers reviewed the coded information and a third reviewer checked the coding for discrepancies thereby, ensuring confirmability or rigour to data analysis [40].

### Participant characteristics

The forum (*n* = 23) included both male (*n* = 14) and female (*n* = 9) adults (ranging in age from 22 to 71 years), including: Community/cultural health workers who work with SSA communities (*n* = 10), SSA leaders/elders (*n* = 7), and SSA community members (*n* = 6). Participants’ ethnic backgrounds were: 91% (*n* = 21) SSA, 4% (*n* = 1) Caucasian, and 4% (*n* = 1) Chinese. These latter two participants were included within the workshop due to their extensive involvement within the community as health workers and were well placed as key informants. Participant educational levels were: university post-graduate (*n* = 3), university graduate (*n* = 8), some university (*n* = 3), diploma (*n* = 3), and high school (*n* = 3). Participants represented a range of geographic locations within Queensland, Australia (Brisbane, Ipswich, Logan, Rockhampton, Toowoomba), and countries of origin (Burundi, Eritrea, Kenya, Rwanda, South Sudan, Tanzania, Zimbabwe).

## Results

Identification of the relationship and commonalities between the participants’ comments at the forum were analysed using thematic analysis and two key themes were identified and three subthemes from each major theme emerged. The major themes that emerged from the data were *Risks Factors for HIV Transmission* and *Prevention*, which were further arranged into subthemes. The subthemes that emerged from Risk Factors for HIV Transmission included: (a) Risks in Australia; (b) Gender, cultural norms and youth; and (c) Risks during return visits to home country. The subthemes that emerged from Prevention included: (a) Barriers to prevention; (b) Prevention during return visits to home country; and (c) The acceptability or effectiveness of PrEP. The major themes and subthemes included factors such as gender, personal and cultural beliefs, age, attitudes, stigma, as well as practices related to sexual and cultural influences. However, participants’ perceptions regarding the transmission of HIV upon settling into Australia was noteworthy and was identified as a significant risk factor.

### Risk factors for HIV transmission

The first theme to emerge from the data related to risk factors associated with HIV transmission secondary to SSA migrants re-settling in Australia. This theme was significant because the majority of participants cited that members of their communities did not feel particularly at risk of acquiring HIV once they lived in Australia. Of note, numerous rationales indicated that SSA migrants considered HIV transmission was no longer relevant because the risks in Australia were perceived as less or non-existent.

#### Risks in Australia

Perceptions that SSA migrants were at less risk of transmitting HIV after relocating to Australia was partly based on the assumption that all SSA migrants had been tested for HIV prior to arrival in Australia. Additionally, because of the low prevalence of HIV in Australia there was the perception that there would be limited transmission of HIV following relocation. Furthermore, psychological and psycho-social processes, such as avoidance of people with HIV, denial that HIV is a problem in Australia, and reduced stigma associated with HIV in Australia meant that there was no longer concern about acquiring HIV. Examples of the perceptions of SSA migrants towards HIV risk in Australia reported at the forum by community members included, “it won’t happen to me”; “…if a person looks healthy, they don’t have HIV”; “people think now they are in Australia, there is no problem…[no] risk”; and “when they immigrated they had testing done and think everyone is tested and OK – no HIV.”

#### Gender, cultural norms and youth

Community members also verbalised that, although SSA migrants were settled in Australia, there remained other risk factors related to HIV transmission. For instance, gender, cultural norms regarding relationships, and age were reported by the participants as factors contributing to greater risk of HIV transmission. Participants perceived there was a greater risk of men contracting HIV due to socio-cultural and historical influences. Moreover, men were viewed as being at greater risk to HIV exposure because males were more likely to adopt SSA cultural sexual norms such as multiple and, concurrent relationships. One participant stated, “men may have a second or third wife, increasing chance of passing on disease” and another claimed, “men are promiscuous and have many partners.” Further, participants frequently commented that there existed a greater cultural acceptance of polygamy and concurrent relationships for some men within SSA communities. Statements made during the forum included, “polygamy is more acceptable and encourages men to have multiple partners” and “having multiple sexual partners…open…secret.” Therefore, despite SSA migrants residing in Australia, risk factors for HIV transmission persist due to specific cultural beliefs and practices.

HIV transmission in Australia was also discussed in terms of young people (adolescents) being at a greater risk due to their propensity to seek greater personal freedom and reluctance to use condoms. There was a shared belief at the forum that life in Australia resulted in young people rejecting or drifting from traditional values and adopting more hedonistic lifestyles. This cultural freedom, combined with the energy and experimentation of youth were viewed as powerful co-factors of risk for acquiring HIV. Conversely, the perception by participants of youth being powerful and rebellious included the perspective that young people were vulnerable victims. Participants reported, “our youth don’t want to wear condoms and they do not want to live at home with parents” and “systems in Australia grant freedom for young people to move away from parents… [there is a] cultural shift…parents feel they don’t have a voice [and] removed power from parents.” As such, adult participants in theforum concluded that the risk of HIV transmission in Australia was primarily a concern for SSA youth (in addition to the risks associated with returning to home countries for visits), which may or may not be consistent with views shared among SSA young people themselves.

#### Risk during return visits to home countries

Forum participants discussed a range of risk factors (specific to both males and females) returning to countries of origin but in general believed that single people were at particular risk from travel because, “single people travel a lot, changed sex partners more, and looked for better sex partners.” Discussion also focused on the role of cultural beliefs and practices associated with the transmission of HIV. When SSA migrants returned to their home country there existed the expectation that cultural rules and traditions re-applied and were reintroduced. Complex cultural rules and traditions regarding social and sexual relationships are powerful influences within SSA communities and to reassimilate with their homeland, certain rituals need to be adhered to upon returning to home countries. Therefore, despite being separated from their country of origin while living in Australia, the need to integrate and blend back into their country strongly impacted on visitors returning to their homeland. Some examples reported by participants of historically embedded cultural practices include, *“*when a man dies the younger brother gets the wife” and “some men are paid by parents to have sex with their young girls as a rite of passage.” Some men reported an awareness of risks for HIV transmission associated with widowhood rites and a desire not to follow cultural practices, “I don’t want an HIV positive wife because it puts me at risk of HIV”, whilst men that have sex with younger females or the wife of a deceased brother “can be open about being HIV positive.”

Some of the participants spoke of particular cultural practices that community members may be more likely to participate in during visits to home countries. These cultural practices may be associated with religious and cultural ceremonies, rites of passage or cultural beliefs, and which were understood by some participants to be relevant to particular areas of SSA. Participants described some of these beliefs from their country of origin as, “sleeping with a virgin or young girl will cure HIV”; “they can treat/cure HIV naturally [without medications]”; and the belief that having sex with young girls can “cleanse you of HIV.” Homeland practices that place visitors at risk for HIV transmission include cultural ceremonies involving “returning for cultural ritual increasing blood risk…may include cutting with razors” and “tribal risks of children travelling back to Africa…even with parents.” However, the most consistent point raised throughout the forum was that men are more at risk of HIV transmission on return visits to their home country due to sexual liaisons resulting from the need or desire to adhere to local cultural practices.

During the forum, the participants frequently discussed that men are most at risk when returning to SSA for visits partly due to access to willing female partners. The perception reportedly held by some local SSA females is that men who have lived in Australia have wealth and power as opposed to people in their homeland. Therefore, SSA men returning home are considered more attractive to females. Similarly, SSA females returning to their homeland were reportedly viewed as *Queens* and sexually desirable by local men. As such, whether male or female, traveling alone and without a partner provides opportunity for sexual liaisons. Comments at the forum included, “men travelling without their wife are sexually active in Africa” and “visitors seen as *Kings* as they have money, [are] treated highly, lots of available sex.” Of note, there exists the “perception that women in [particular country named] are *really good* and you must have sex before you come home, people might think there is something wrong with you if you don’t [have sex]” and that, “men when travelling home are free to play around” and “attractive women are tempting.” One participant summarised these views by saying that on returning to SS Africa “men are considered wealthy and have more women and are tempted to do silly stuff...play around…have lots of unprotected sex.”

Finally, some participants claimed that SSA men returning to their home country were considered “attractive because local females foresaw an opportunity to leave Africa.” Therefore, female participants at the forum frequently discussed the notion that single females living in SSA sought long-term partners with men returning to visit home countries and would use pregnancy to acquire a husband. Comments made included, “women are at higher risk” and “visitors are targeted by women to become pregnant in the belief it opens up a path to Australia.”

The findings presented within the major theme of Risk Factors for HIV Transmission are concerning and of salient public health interest. Participants identified multiple and varied reasons why higher rates of HIV transmission are reported amongst SSA migrants, which may be specific to contexts regarding travel to home countries and within Australia. The lack of awareness that HIV transmission remains a risk in Australia, combined with cultural and gender-ascribed practices, adds additional complexities. Furthermore, the risks of HIV transmission during return visits to home country raise considerable concerns regarding prevention in Australia and in SSA.

### Prevention

Strategies to improve HIV prevention were discussed at length during the forum resulting in Prevention being the second major theme to emerge from the data. A significant topic about prevention related to the responsibility for HIV testing amongst SSA community members as *patients* versus *doctors*. There was a strong belief that it was the doctor’s role to initiate any discussions around HIV testing. Members of the forum spoke of the responsibility and expectation they had of doctors to raise HIV testing with their patients rather than wait for SSA community members to initiate such discussions. Some participants at the forum believed that HIV testing should be conducted as a routine and without consent, in order to reduce late diagnoses and prevent onward transmission. As one participant reported, “it is difficult for us to ask, the tests should be done automatically, not us asking” and “in Africa, the doctors test for everything, consent is not necessary.” Furthermore, forum participants considered the Australia health care system as preferring short consultations and quick throughput, mitigating against more meaningful and comprehensive conversations and broader interventions. Participants commented that there is “difficulty adapting to differences in medical system” between Australia and SSA. Comments that typified this difference included, “In Africa the doctor listens and takes blood and other samples, consent is not required like it is in Australia. You are tested for everything in Africa. In Australia, we only get five to ten minutes and we have to ask the doctor to test us”; and the “Government needs to change policy for GPs [doctors] so they can spend more time with patient.”

Observations about doctors “having five to ten minute consultations” were predominant in discussions about barriers to prevention and there was concern expressed about the need for prevention during return visits to homelands and the acceptability or effectiveness of PrEP. Overall, the subthemes of Prevention incorporated many key barriers to HIV prevention such as stigma, cultural shame and condom use, and limited health education.

#### Barriers to prevention

A number of factors were identified at the forum as to creating a barrier to HIV prevention strategies. For instance, a reluctance to communicate concerns about HIV risks and transmission due to the perceived stigma associated with HIV and cultural shame dominated discussions. Community members perceived that the stigma associated with HIV not only hindered health promotion efforts but also limited any support for SSA people living with HIV. Moreover, participants revealed that the stigma associated with HIV transmission created a strong focus on confidentiality and privacy. The stigma associated with HIV contributed to heightening fear regarding HIV that directed discussions and prevention efforts *underground* within the community. There was a sense that greater openness about HIV would enhance greater acceptance of education. Comments made at the forum revealed that the stigma of HIV “impacts on education, [people] don’t want to talk about sex or condoms” and the lack of education “encourages not using condoms.” However, forum participants tended to agree that, “confidentiality and being private led to greater stigma” and was perceived by the participants that, “HIV posters and health promotion can make HIV positive people feel bad.” A general view held by participants included, “When HIV is disclosed we can help them, people will be free, stigma will go” with open discussion.

Reluctance within the SSA community to promote or engage in open discussion about the impact of HIV was identified as a key barrier to effective prevention. Additionally, shame surrounding discussions about HIV appeared to be closely linked to cultural inhibitions concerning any discourse on sex. For instance, participants stated, “people are reluctant to find support… only [discuss] with close friends, not in public” and there exists “cultural shame to talk about condoms and HIV.” Further, “there is a cultural taboo regarding talking about sex with certain people…one example [a] father-in law should not speak to son-in law about sex with his wife.” As such, community education strategies to prevent HIV transmission would not necessarily occur through open dialogue although some participants suggested that reinforcing messages about personal responsibility and explicit community assurances that HIV is preventable could be beneficial. Participants at the forum commented, “you can be safe if you take precautions” and “HIV is not an accident, it can be prevented.”

#### Prevention during return visits to home country

A strong sub-theme that emerged in the discussions concerning Prevention embraced the need to reduce HIV transmission during return visits to homelands. Prevention initiatives during return visits to Africa focussed on health education and advice, condom use, HIV testing and PrEP. Overwhelmingly, there was strong support during the forum that information and advice needed to be provided to travellers on the risks of HIV transmission when returning to home countries. Of note, participants did not specifically state who (i.e., what roles) should be responsible for disseminating this information to prospective travellers or how this information could be effectively communicated. There was an acknowledgment of the unwillingness within the community to discuss sexual health issues and risks in relation to return visits among many SSA community members because “people don’t talk about sex or HIV prior to travelling back home.”

Forum participants perceived that the provision of more substantial education and increased supply/access to condoms was only a starting point. Information should be provided about “HIV rates for the country they are visiting and the level of risk” and “travellers should be provided with counselling before they leave Australia” and most importantly, “condoms should be put on the list of items when you pack your bags to travel.” Condoms were highlighted as a means of preventing HIV during return visits to home country despite admissions that they were often not used and that there was a general lack of awareness about appropriate condom size and access to female condoms. Participants commented that, “condoms are there, but they are not used… hotel rooms in Africa have condoms in drawers”, but there exists a “lack of awareness that different sizes are available.” Despite the availability of condoms in Africa the more significant issue identified was the reluctance to wear a condom due to a perceived loss in sensation, summarised by one participant as, “I don’t want to eat a lolly with a wrapper on it.” Similarly, there was “negative feedback on female condoms” and there exists a “lack of availability of female condoms.” Being informed about the need for condom use prior to returning to Africa was a significant factor in relation to prevention but equally noteworthy was the potential for use of prophylaxis medication such as PrEP.

#### The acceptability or effectiveness of PrEP

PrEP has been identified as an important prevention strategy for high risk SSA migrants [6, 43, 44]. When participants were asked to discuss their views of the acceptability or effectiveness of PrEP within the community practical concerns such as cost, the requirement for daily medication and regular monitoring were raised as potential burdens. Comments made by participants included, “it would be hard if costs were involved as lots of people are struggling financially and to ask for them to pay for something else”; “three monthly check ups is difficult, people might not have the time or want to do this” and it wasn’t known “how to monitor if medication is effective?”

There were also concerns regarding the need to overcome stigma specifically in relation to PrEP. The use of PrEP within the community was discussed through a moral lens, indicative of particular behaviours, attitudes and lifestyle. For instance, there is a “stigma of taking PrEP, as the community might think you are a commercial sex worker” and there is a “potential stigma from taking a pill every day as there would be gossip and could cause trouble within families and the community…everyone would know”; “PrEP might be linked to commercial sex work or male being promiscuous and this would have a negative impact. African communities are complex.” The overall view was that, “many people will not think it is helpful, they will be embarrassed, people will ask if you are a sex worker.” Additionally, “if the man is on the pill they will think he is playing around” and “if wife finds out she will think he is playing around and then it will be spread to the community.” In general, it was perceived that the stigma of taking PrEP would invite judgement from the community and would therefore pose a formidable barrier to PrEP’s acceptance as a mainstream prevention strategy.

Other concerns expressed regarding potential government monitoring of PrEP users related to the possible side effects and lack of willingness to participate in any PrEP research trials. Overall, forum participants displayed both reluctance and curiosity about PrEP, which was fuelled by a mixture of suspicion towards government control, potential harms, and fear of communal approbation. Statements to support the inappropriateness of PrEP for SSA people included, “This is not appropriate for our culture. There are many things to consider. We are different to Australians and Europeans” and “cultural background impedes the use of services [and] religious beliefs.” Another noteworthy factor surrounded clinical trials and that the “African community [is] tired of always being a target of research, they are sceptical” and “if involved in the *trial*, who would be responsible for adverse side effects?” Participants voiced that the community would want more information such as, “will there be a *register*… for people taking PrEP?” and “how to monitor if medication [PrEP] is effective?” Further, concerns were raised as to “who is liable for the risk of participating in a trial of this drug? What happens if there are ongoing side effects? What are the long-term side effects?”

Although there was the view that, “PrEP use may encourage more sex, encouraging less condom use”, there was also support generated from community members for the concept of PrEP as a prophylaxis, in situations such as sero-discordant relationships. However, it was also noted that how this strategy could be effectively promoted and implemented was unclear. Although, it was understood that, “PrEP would be useful for HIV negative man if his wife was positive”, issues were raised as to how information could be effectively disseminated across the communities. Moreover, there is a “need for information to be presented in community language” and “there are 54 countries with different cultures and communities so a community workshop is needed for each different culture and community… some communities are more complex.”

Many suggestions were made regarding who may be best placed to promote PrEP and in what forums or contexts education could be conducted in these communities. As with other discussions, the role of the doctor or other clinician as *expert* was emphasised, with community workers offering a more supportive role. For example, “community leaders [should] organise workshops, with experts present and have translators as well. Expert can answer questions, as community leaders don’t have the expertise and it is too much to expect them to take the message back themselves. Training [is needed] for community leaders on awareness sessions very important…education for community leaders.” Additionally, “social workers should attend these workshops” and “medical doctors may be the best people to take message to community with community leader support – experts need to explain side-effects.” A final comment made was, “community workers can pass on the simple message of PrEP [as a potential preventive strategy] and leave medical experts to explain complexities of treatment, cost and timing of 3 monthly check-ups.” A further sentiment expressed by one participant was that, “initiatives should come from those who are HIV positive.”

### Summary

Thematic analysis of the data collected at the community forum revealed there are complex cultural beliefs, rituals and practices that need to be addressed if HIV transmission within the SSA community is to be prevented. Relocation to Australia from SSA does not resolve the issues associated with HIV transmission and risk factors continue to exist once people have resettled not only in Australia but more so, when SSA migrants return to their home countries. Prevention remains critical and identifying acceptable strategies requires further exploration.

## Discussion

Although this community forum was not necessarily representative of the general SSA Australian community or the entire community leadership within Queensland, it did provide an opportunity for key stakeholders to discuss contemporaneous issues surrounding HIV transmission. Data from the community forum enabled a wide range of personal and cultural beliefs and practices to be identified, which significantly influence HIV risk transmission. Furthermore, the participants’ views of prevention of HIV in Australia and during return visits to home countries was noteworthy. Forum discussions highlighted the significant transmission risk regarding international travel for settled SSA Australian migrants travelling for short periods to home countries and the potential barriers to effectively reduce risks. Fakoya and colleagues [2] conducted a systematic review investigating post-migration acquisition of HIV in migrants from high prevalence countries (Africa/SSA, Asia and Caribbean) now residing in Europe. They found evidence of post-migration acquisition of HIV in all studies with acquisition rates for SSA migrants ranging from 2% in Switzerland to 29% in France. These findings emphasise the increased transmission risk for this population.

Forum participants also stressed the ongoing influence of traditional cultural norms on sexual and relationship attitudes and practices in Australia, with particular relevance to HIV transmission and prevention. Individualised risk reduction strategies were necessary for each context and would require a community effort, drawing upon the cultural authority of the leadership, the professional authority of doctors, and the experiential authority of HIV affected persons to engage the SSA communities in a meaningful dialogue.

Throughout the discussions denial, perceptions of low HIV risk, stigma and cultural norms were the salient barriers to HIV prevention efforts. Low levels of perceived HIV risk has been reported as a barrier to HIV testing [45] and to the uptake of PrEP [46]. The perception of low risk within the community was reinforced by assumptions that immigration based HIV testing and low HIV prevalence within Australia, prevented HIV transmission amongst SSA migrants. It has been suggested once migrants from high prevalence countries have lived in a low prevalence country for many years they may develop a perception of low HIV risk, which may be very different from their actual behavioural HIV risk [47].

A number of other factors were discussed that promoted HIV transmission, such as cultural, historical, gender and social beliefs, and sexual freedom during return visits to home countries. Further, Australian lifestyle, reluctance to consider the impact of gender and traditional relationship norms upon HIV risk and prevention were significant issues.

Stigma was similarly fuelled by perceptions of strict confidentiality rendering HIV invisible within the community, the reluctance of affected individuals and family to speak out and engage the community, the reluctance of the community to discuss its role in perpetuating silence, and cultural, historical and social norms that associated HIV with moral culpability, death and disease. Stutterheim and colleagues [48] found HIV stigmatising beliefs were strengthened and prolonged by “silence, denial and [cultural] taboo” (pp. 478) prohibiting talk about sex and HIV. A study conducted in Australia found social and cultural taboos, such as discussing sex, as a barrier to seeking information about HIV [30]. Agu and colleagues [49] investigated migrant help-seeking in relation to experienced stigma and discrimination and found the experience of stigma developed in home countries continue to pervade the expectation of stigma once resettled, suggesting past/historical experiences of stigma continue to be a significant barrier to help-seeking behaviour [50].

With respect to mitigating risks associated with international travel, a clear role for education, condom promotion and testing was articulated. Central to this, was the role of the local doctor or health worker as counsellor, educator and clinician. A difference in expectations was identified with community members stressing it was the responsibility of their doctors to initiate HIV testing as required, rather than a patient-directed approach. Furthermore, counselling and testing for HIV and other STIs should be considered routine prior to and on return from overseas travel. Poor health literacy and uncertainty as to how to navigate a different health system can be overwhelming for migrants, where cultural norms and language, pose significant barriers [51].

Whilst there was consensus for traditional forms of HIV prevention, education, counselling, testing and condom promotion to be normalised within the community, the role of PrEP was controversial and met with degrees of scepticism, uncertainty, interest and hope. Certainly, formidable cultural barriers fostered by particular social/historical/political experiences within the SSA context need to be addressed in a more culturally-responsive manner to ensure a level of acceptability within the community. Fear of identifying as a sex worker, sexually adventurous or unfaithful was strongly voiced in this forum, coupled with an underlying suspicion of any new innovation.

### Limitations

The limitations of recording and analysing data from a community forum such as this are self-evident. There was substantial content discussed during informal or small group discussions outside of the focussed discussions (e.g., during breaks) that could not be captured and would have provided rich context to the formal content. Any small group of self-selecting participants carries significant limitations for community representation, particularly those who may have difficulties speaking English, or lacked the resources or time to attend a community event held on a weekend. Consequently, young people and women were underrepresented. It should be noted that information about young people was provided by adults and not young people, per se, and represents a useful target to include young people in future similar research to speak on behalf of themselves. Also, as participant characteristics regarding socio-demographic features of participants (e.g., education level, religious background), there may be limitations in representativeness of the cohort and generalisability of findings, which is recommended to be included in future research. Further, the diversity of SSA communities, the plurality of views, traditions and beliefs within each country and across countries, will confound any attempt to draw generalisations from a small group. It must be acknowledged that participants would express views that were not representative of other participant’s experiences, nor of their own countries of origin. Consequently, it was important to seek commonalities across themes whilst recognising the relativity and personal bias of each person’s perception. Further, due to the nature of the community forum-based discussion, it remains unknown the extent to which participants were sharing information on behalf of their own personal experiences versus view held among other community members, and would be a useful consideration for future research.

Discussions were held in small groups and within the greater group, and always as mixed gender; which may have impacted willingness to share information in a mixed-gender forum. It may have been more culturally appropriate to facilitate same gender groups to ensure all voices could be adequately heard. Certainly, individual interviews would have promoted more disclosure and detailed information, allowing for more analysis of issues, however open discussion between people provided an opportunity for new ideas to be exchanged. The gender and ethnicity of the participants, facilitators or scribes (some not from a SSA background) may also influence responses; however, the ability of individuals to speak as a representative for their communities may have allowed for more free expression rather than reflecting on solely personal experiences per se. The verbatim scribing of group discussions as opposed to recorded transcripts would certainly have impacted the detail, quality and nuances of content. Nonetheless, responses may have been more candid for participants knowing responses were not electronically recorded, especially due to concerns voiced of being an over researched group and expressed scepticism relating to research.

### Implications

The feedback collected from this forum of SSA Australians has certainly indicated the need for further assessment and scoping of PrEP as an appropriate and feasible intervention within the community with careful attention given to the negative community perceptions and its potential impact on individuals, against potential benefits and strategies to more effectively promote cultural responsiveness. The reference to *trials* when describing innovative medical programs such as PrEP can create a level of anxiety and suspicion concerning research *on the community*. A recent PrEP trial in South Africa conducted by Van Der Straten and colleagues [52] explored the socio-cultural influence on self-reported PrEP adherence and found ambivalence towards research was one of three main themes that impacted their trial. Geissler and Pool [53] suggest the African cultural norm of *rumours* are a means to perpetuate ambivalence towards research which has a history related to colonialization trauma and social relations with medical research. They urge researchers to be mindful of this and maintain an open dialogue with the community. As with any PrEP program, extensive education regarding appropriate use, side effects, and limitations will need to be applied diligently in order to overcome myths and misconceptions.

Development of more culturally appropriate models of health promotion and care will need to account for the unique historical, social, religious and traditional context of each of our SSA Australian communities, particularly the role of stigma and how it may limit access to testing, treatment, education and preventive technologies. The cultural and social expectations placed upon people returning to home countries and their capacity to increase or mitigate risk in high prevalence settings needs further study. Additionally, the role of trauma for those migrating from zones of high conflict or displacement needs to be better understood, as this may contribute to greater suspicion and reluctance. Additional factors, beyond the scope of this project are also known to impact upon HIV transmission and prevention approaches, including cultural practices (e.g., genital mutilation; [54]), individual beliefs and risk perceptions regarding HIV transmission (e.g., a belief that one cannot acquire HIV if circumcised as it is an “invisible condom”; pp. 6; [55]), religious beliefs (e.g., for men being married to more than one wife is permissible within the Muslim faith and cultural practice of polygamy; [56]), and the extent of acculturation and assimilation to practices within destination country versus home country, which contribute to the complexities for health promotion within SSA communities and represent important areas to explore in future research.

Cultural and historical experiences shape a community’s attitudes and understanding of the role of research and the medical profession, particularly that of the doctor. Concepts such as consent, privacy, confidentiality and autonomy can have very different meanings across different populations. Certainly, the role of the doctor as a proactive initiator of health service provision rather than a responsive provider of client driven services needs to be better acknowledged. The additional need for cross cultural training and integrated culturally responsive care, facilitated through community workers, cultural peer navigators and translators, would appear to be critical for HIV intervention strategies such as early testing and treatment and PrEP, to be effective. Further, it is essential that doctors working with these communities are aware of HIV risks and prevention approaches and respond in a culturally responsive manner [57].

## Conclusions

Our forum of key community stakeholders has identified a wide range of personal and cultural beliefs and practices that have an explicit and implicit effect upon HIV transmission rates and use of prevention strategies. In particular, the role of international travel as a risk factor for HIV acquisition needs better analysis, as do the role of the doctor and PrEP as responses to that risk.

## Abbreviations

CALD: Culturally and linguistically diverse
HIV: Human immunodeficiency virus
NGO: Non-government organisation
PrEP: Pre-exposure prophylaxis
SSA: Sub-Saharan Africa
